# Backbone and methyl side-chain resonance assignments of the Fab fragment of adalimumab

**DOI:** 10.1007/s12104-024-10187-1

**Published:** 2024-06-26

**Authors:** Muzaddid Sarker, Yves Aubin

**Affiliations:** 1 Centre for Oncology, Radiopharmaceuticals and Research, Biologics and Radiotherapeutic Drugs Directorate, Health Canada, 251 Sir Frederick Banting Driveway, Ottawa, ON K1A 0K9 Canada; 2 Department of Chemistry, Queen’s University, 90 Bader Lane, Kingston, ON K7L 3N6 Canada; 3 Department of Chemistry, Carleton University, 1125 Colonel By Drive, Ottawa, ON K1S 5B6 Canada

**Keywords:** Monoclonal antibody, Adalimumab, NMR spectroscopy, Fragment antigen binding, *Escherichia coli*

## Abstract

Adalimumab is a therapeutic monoclonal antibody developed to target human TNF an important mediator of immune-mediated inflammatory diseases such as rheumatoid arthritis, amongst others. The 48 kDa Fab fragment of adalimumab was produced in *Escherichia coli* using a single chain approach to allow complete isotopic incorporation of deuterium, carbon-13 and nitrogen-15 along with the protonated isoleucine-d, valine and leucine methyl groups. Here we report the near complete resonance assignment of the polypeptide backbone and the methyl groups of isoleucine, leucine and valine residues.

## Biological context

### Brief biology on TNF

Tumor necrosis factor (TNF) also known as TNF-alpha is a cytokine released from cells as a soluble trimeric protein (sTNF) from its transmembrane form (tmTNF) by TNF-alpha-converting enzyme (TACE). When it is present at low concentrations, it stimulates host defense mechanisms to fight infections that may follow organ injury. However, at high concentrations, it can promote inflammation and organ injury (Tracey et al. 2008). Other cytokines, such as interleukin-1and interleukin-17 also have proinflammatory properties. Abnormal or excessive production of TNF can induce a variety of immune-mediated inflammatory diseases such as rheumatoid arthritis (RA), inflammatory bowel disease (IBD) psoriatic arthritis (PsA), psoriasis (PS) ankylosing spondylitis (AS), and non-infectious uveitis (NIU). TNF can both mediate a variety of direct pathogenic effects and induce the production of other mediators of inflammation (interleukins and other cytokines) and tissue destruction. TNF is an important cytokine at the top of the inflammatory cascade as well as part of an intricate pro-inflammatory network of cytokines (Tracey et al. 2008).

The first TNF antagonist biologic drug was a monoclonal anti-TNF antibody named infliximab (brand name Remicade®). Many studies (Choy and Panayi 2001; Tracey et al. 2008; Jang et al. 2021) using this mAb confirmed the central role of TNF in the complex biology of the diseases mentioned earlier. Infliximab is a chimeric therapeutic mAb where the antigen binding fragment (Fab) has its variable domains derived from mouse while the constant regions are human. Such chimeric mAbs have a higher tendency to elicit the production of neutralizing antibodies that, with time, will render the drug less or not effective at all. Adalimumab (brand name Humira® (Abbvie), and its biosimilars Amgevita (Amgen); Cyltezo (Boehringer Ingelheim); Abrilada (Pfizer); Imgraldi (Samsung) and more that are in clinical trials) is a human mAb of the immunoglobulin G1 (IgG1) class and was developed to alleviate (mitigate) this problem. Here we present the backbone and side chain methyl group of isoleucine, leucine and valine residues chemical shifts of the single chain Fab fragment of adalimumab.

## Methods and experiments

### Expression and purification of adalimumab-Fab

The amino acid sequence used for the production of samples of labelled adalimumab-scFab has been described in details previously (Gagné et al. 2023). The heavy chain of adalimumab Fab domain and a portion of the hinge region (see (Hodgson et al. 2019) for complete amino acid sequence), residues Glu1 to Pro234 where Cys233 has been mutated to Ala233, was linked to residues Asp1 to Cys214 of the light chain via a linker made of five (GGGGS) elements plus SSGLVPRGS. The last residues of the linker contain a thrombin recognition site (LVPRGS). A poly-histidine tag (MGSSHHHHHH HHHHSSGHMLVPRGS) was fused to the amino terminal of this polypeptide. Papain cleavage, normally takes place between His2289 and Thr229, completely removed the fusion tag and the linker while leaving the thrombin recognition (LVPRGS) site at the N-terminal end of the light chain at the expense of great sample loss (80–90%). Therefore, we initially carried out the backbone and side-chain assignment of histag-adalimumab-scFab fragment, then produced a cleaved sample for final resonance assignment with the following sequence, where the extra residues (LVPRGS) were numbered starting at (−5):


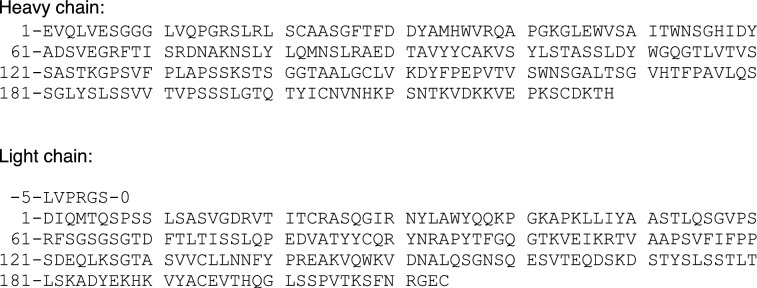


Expression of labeled ^2^H–^13^C–^15^N-histag-adalimumab-scFab was carried out exactly as described previously (Gagne et al. 2024) with the exception that cells were harvested after 18 h post induction. The sample for resonance assignment contained 145 µM ^2^H–^13^C–^15^N-adalimumab-Fab (7 mg/mL) in 20 mM sodium acetate-d3 at pH 5.0 with 5% v/v deuterium oxide for lock frequency purposes in 50 µL and transferred in a 1.7 mm tube. Production of labeled ^2^H–^13^C–^15^N–^1^H–methyl-(Ile, Leu, Val)-histag-adalimumab-scFab was conducted as described previously (Gagne et al. 2024). The final sample contained 400 µM ^1^H–I(d1)LVmethyl-histag–^2^H–^13^C–^15^N-histag-adalimumab-scFab in 300 µL (5 mm Shigemi tube) in the same buffer.

## NMR experiments

Data were collected at 40 ℃ (313 K) on Bruker AVANCE NEO 600 MHz (side-chain methyls assignment), AVANCE III-HD 700 MHz (backbone assignment) and AVANCE II 900 MHz NMR spectrometers equipped with 5 mm, 1.7 mm, and 5 mm, respectively, TCI cryogenically cooled triple resonance inverse probeheads fitted with z-axis gradients. Chemical shift resonances were referenced with sodium 2,2-dimethyl-2-silapentane-5-sulfonate (DSS). Data collection for the assignment of the backbone and side chain methyl resonances was carried out exactly as described previously using the same pulse program and parameters (Gagne et al. 2024).

## Data analysis and resonance assignment and validation

NMR data were processed using nmrPipe (Delaglio et al. 1995). Sequential assignment was carried out manually using POKY (Lee et al. 2021).

## Validation of NMR assignment

The web server of I-PINE (http://i-pine.nmrfam.wisc.edu/index.html) (Lee et al. 2019) was used initially through the POKY interface to verify and validate the adalimumab-Fab assignments. All peak lists from TROSY-based NMR experiments, namely 2D-^15^N-HSQC, 3D-HNCO, HN(CA)CO, HNCA, HN(CO)CA, HNCACB, and HN(CO)CACB, were used as input files. Analysis of the I-PINE output showed near-complete correctness of the manual assignments and identified a very few new assignments. Upon completion of manual assignments, we tested the new assignment protocol BARASA (Bishop et al. 2023) running under NMRBox using the same peak list and assignments as input data used for I-PINE. A total of 3 BARASA rounds were conducted, using 80 concurrent threads with 0.99 convergence p-value, and a stepwise energy drop of −500. Ca, Cb, and CO zero points were all set to 0.20, with a chemical shift energy range of −100 to 100.

## Extent of assignments and data deposition

### Resonance assignment of backbone atoms

We compared the 2D–^1^H–^15^N HSQC of histag-adalimumab-scFab with the fully cleaved adalimumab-Fab. The extra resonances belonging to the histag and linker were well resolved from any backbone resonances of the Fab while all backbone resonances of both samples were overlapping with each other, indicating that the assignment of the histag-scFab can be directly used for the Fab. Considering that the 1.7 mm cryoprobe only requires a very small sample size (40 mL), we carried out resonance assignment of the backbone resonances on a sample of ^2^H–^13^C–^15^N-adalimumab-Fab. On the other hand, side-chain methyl assignment was carried out using an uncleaved sample. The Fab fragment contained a total of 442 amino acids (47.7 kDa) with the heavy chain having 228 residues (11 prolines) and the light chain having 214 residues (11 prolines).

**Fig. 1:**
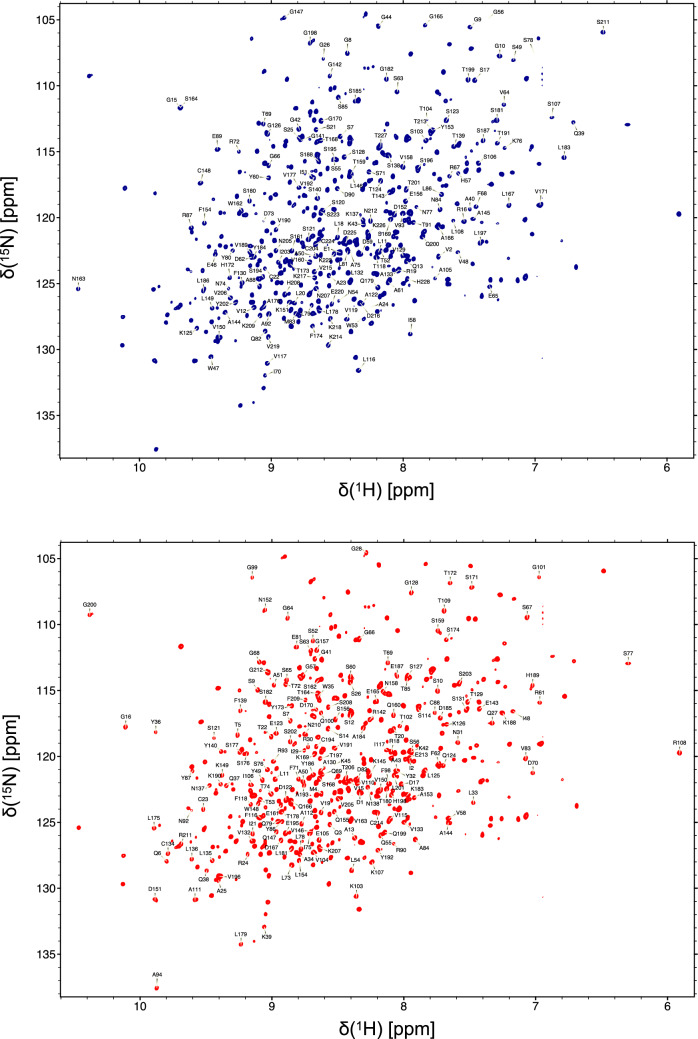
Two-dimensional ^1^H–^15^N-TROSY-HSQC spectrum of ^2^H–^13^C–^15^N-adalimumab-Fab acquired at 900 MHz at 40 ℃. The backbone amide chemical shift assignment of heavy and light chains is indicated in the blue and red spectra, respectively

The two-dimensional TROSY-HSQC spectrum of ^2^H–^13^C–^15^N-adalimumab-Fab shows well-dispersed resonances, typical of well-folded Fab (Fig. 1). A total of 381 (90.7%) ^1^H–^15^N backbone peaks were assigned, with 183 (84.3%) and 198 (97.5%) in the heavy and light chains, respectively. Assigned carbons include 407 (92.1%) ^13^C–O, 407 (92.1%) ^13^Cα, and 376 (91.7%) non-glycine ^13^Cβ. The Fab fragment is composed of four immunoglobulin domains that are each stabilized by one disulfide bond: Cys22-Cys96 (heavy chain, VH), Cys148-Cys204 (heavy chain, CH1), Cys23-Cys88 (light chain, VL), Cys134-Cys194 (light chain, CL), and one bond that links the heavy to the light chain Cys224-Cys214. Analysis of the cysteine Cβ chemical shifts indicates that their values (higher than 35 ppm) is consistent with properly formed disulfide bond. (Schulte et al. 2020).

### Resonance assignment of isoleucine *delta*-1, leucine and valine methyl groups

**Fig. 2:**
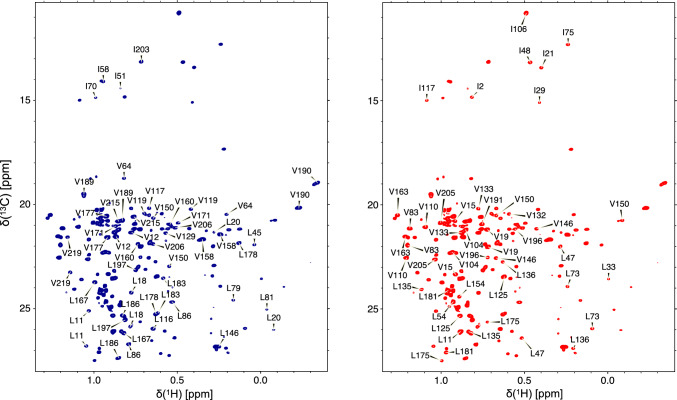
Two-dimensional ^1^H–^13^C CT-HSQC spectrum of the methyl spectral region of histag-adalimumab-scFab. The spectrum was acquired using a ^1^H–I(δ1)LVmethyl–^2^H–^13^C–^15^N-histag-adalimumab-scFab sample, with acquisition at 40 ℃ and 1 GHz, with 28 ms constant-time delay. The methyl chemical shift assignment of isoleucine-δ1, leucine, and valine of heavy and light chains is shown in the blue and red spectra, respectively

Using the carbon tocsy versions of experiments, a total of 11 isoleucines (100%), 27 leucines (88%), and 30 valines (81%) were assigned (Fig. 2). Only 4 leucines have not been assigned, (Leu4, Leu108, Leu132 of the heavy chain, and Leu46 of the light chain), and 7 valines (Val5, Val37, Val 99, Val129, Val192 heavy chain and Val58 of light chain).

## Comparison with trastuzumab-scFab and NISTmAb-Fab assignments

Adalimumab, trastuzumab and the NIST-mAb are three monoclonal antibodies of the IgG1 class with light chain kappa with identical amino acid sequences in their constant heavy 1 (C_H_1) and constant light domain (C_L_). As reported earlier (Gagne et al. 2024), we compared the assignment of these domains of trastuzumab-scFab the yNISTmAb-Fab (Solomon et al. 2023) that were overlapping to validate our assignments.

## Validation of the backbone assignment

I-PINE was first used to validate the manual assignment and to help in identifying non-assigned residues. Out of the total 442 residues of adalimumab Fab, we initially manually assigned 395 residues corresponding to a coverage of 89.4%. I-PINE assigned 401 residues (i.e., only a few extra) and all residues except one of our 395 manual assignments matched the I-PINE results.

We also used BARASA for our manual assignment validation and identification of non-assigned residues. BARASA predicted assignments of 417 residues and we were able to assign additional residues, namely Asn92, Arg93, Ala94 of the light chain and Thr52, Trp53, Asn54, Ser55, Gly56, Ser78, Ser194 of the heavy chain. We also corrected assignments of Val58 of light chain using BARASA. The third round of BARASA confirmed correctness of all assignments.

## Outlook

The resonance assignment of adalimumab will provide a powerful tool for the characterization of Humira® and its biosimilars. In addition, it will facilitate the application of NMR spectroscopy techniques to probe protein dynamics, epitope binding and drug excipients interactions at atomic level.

## Data Availability

Chemical shifts and Bruker raw data *ser* files were deposited in the BMRB data bank with entry number 52274.

## Abbreviations

mAb: Monoclonal antibody.
scFab: Single-chain fragment antigen-binding.
Fab: Fragment antigen-binding
VH: Heavy chain variable domain.
VL: Light chain variable domain

## References

[CR1] Bishop AC, Torres-Montalvo G, Kotaru S, Mimun K, Wand AJ (2023) Robust automated backbone triple resonance NMR assignments of proteins using bayesian-based simulated annealing. Nat Commun 14:1556. 10.1038/s41467-023-37219-z 36944645 10.1038/s41467-023-37219-z PMC10030768

[CR2] Choy EHS, Panayi GS (2001) Cytokine pathways and joint inflammation in rheumatoid arthritis. N Engl J Med 344:907–916. 10.1056/NEJM200103223441207 11259725 10.1056/NEJM200103223441207

[CR3] Delaglio F, Grzesiek S, Vuister GW, Zhu G, Pfeifer J, Bax A (1995) NMRPipe: a multidimensional spectral processing system based on UNIX pipes. J Biomol NMR 6:277–293 8520220 10.1007/BF00197809

[CR4] Gagné D, Sarker M, Gingras G, Hodgson DJ, Frahm G, Creskey M, Lorbetskie B, Bigelow S, Wang J, Zhang X et al (2023) Strategies for the production of isotopically labelled Fab fragments of therapeutic antibodies in *Komagataella phaffii* (*Pichia pastoris*) and *Escherichia coli* for NMR studies. PLoS ONE 18:e0294406. 10.1371/journal.pone.0294406 38019850 10.1371/journal.pone.0294406 PMC10686436

[CR5] Gagne D, Aramini JM, Aubin Y (2024) Backbone and methyl side-chain resonance assignments of the single chain Fab fragment of trastuzumab. Biomol NMR Assign. 10.1007/s12104-024-10177-3 10.1007/s12104-024-10177-3 PMC11511688 38717571

[CR6] Hodgson DJ, Ghasriani H, Aubin Y (2019) Assessment of the higher order structure of Humira®, Remicade®, Avastin®, Rituxan®, Herceptin®, and Enbrel® by 2D-NMR fingerprinting. J Pharm Biomed Anal 163:144–152. 10.1016/j.jpba.2018.09.056 30296716 10.1016/j.jpba.2018.09.056

[CR7] Jang D-I, Lee AH, Shin H-Y, Song H-R, Park J-H, Kang T-B, Lee S-R, Yang S-H (2021) The role of tumor necrosis factor alpha (tnf-α) in autoimmune disease and current TNF-α inhibitors in therapeutics. Int J Mol Sci. 10.3390/ijms22052719 10.3390/ijms22052719 PMC7962638 33800290

[CR8] Lee W, Bahrami A, Dashti HT, Eghbalnia HR, Tonelli M, Westler WM, Markley JL (2019) I-PINE web server: an integrative probabilistic NMR assignment system for proteins. J Biomol NMR 73:213–222. 10.1007/s10858-019-00255-3 31165321 10.1007/s10858-019-00255-3 PMC6579641

[CR9] Lee W, Rahimi M, Lee Y, Chiu A (2021) POKY: a software suite for multidimensional NMR and 3D structure calculation of biomolecules. Bioinformatics 37:3041–3042. 10.1093/bioinformatics/btab180 33715003 10.1093/bioinformatics/btab180 PMC8479676

[CR10] Schulte L, Mao J, Reitz J, Sreeramulu S, Kudlinzki D, Hodirnau VV, Meier-Credo J, Saxena K, Buhr F, Langer JD et al (2020) Cysteine oxidation and disulfide formation in the ribosomal exit tunnel. Nat Commun 11:5569. 10.1038/s41467-020-19372-x 33149120 10.1038/s41467-020-19372-x PMC7642426

[CR11] Solomon TL, Chao K, Gingras G, Aubin Y, O’Dell WB, Marino JP, Brinson RG (2023) Backbone NMR assignment of the yeast expressed Fab fragment of the NISTmAb reference antibody. Biomol NMR Assign 17:75–81. 10.1007/s12104-023-10123-9 36856943 10.1007/s12104-023-10123-9

[CR12] Tracey D, Klareskog L, Sasso EH, Salfeld JG, Tak PP (2008) Tumor necrosis factor antagonist mechanisms of action: A comprehensive review. Pharmacol Ther 117:244–279. 10.1016/j.pharmthera.2007.10.001 18155297 10.1016/j.pharmthera.2007.10.001

